# Requirement of Podocalyxin in TGF-Beta Induced Epithelial Mesenchymal Transition

**DOI:** 10.1371/journal.pone.0018715

**Published:** 2011-04-12

**Authors:** Xiaobo Meng, Peyman Ezzati, John A. Wilkins

**Affiliations:** 1 Manitoba Centre for Proteomics and Systems Biology, Department of Internal Medicine, University of Manitoba, Winnipeg, Manitoba, Canada; 2 Biochemistry and Medical Genetics, University of Manitoba, Winnipeg, Manitoba, Canada; University of Colorado, Boulder, United States of America

## Abstract

Epithelial mesenchymal transition (EMT) is characterized by the development of mesenchymal properties such as a fibroblast-like morphology with altered cytoskeletal organization and enhanced migratory potential. We report that the expression of podocalyxin (PODXL), a member of the CD34 family, is markedly increased during TGF-β induced EMT. PODXL is enriched on the leading edges of migrating A549 cells. Silencing of podocalyxin expression reduced cell ruffle formation, spreading, migration and affected the expression patterns of several proteins that normally change during EMT (e.g., vimentin, E-cadherin). Cytoskeletion assembly in EMT was also found to be dependent on the production of podocalyin. Compositional analysis of podocalyxin containing immunoprecipitates revealed that collagen type 1 was consistently associated with these isolates. Collagen type 1 was also found to co-localize with podocalyxin on the leading edges of migrating cells. The interactions with collagen may be a critical aspect of podocalyxin function. Podocalyxin is an important regulator of the EMT like process as it regulates the loss of epithelial features and the acquisition of a motile phenotype.

## Introduction

Epithelial mesenchymal transition (EMT) is characterized by a loss of the epithelial cell properties of apical basal polarity and tight cell – cell adhesions. These are accompanied with the acquisition of mesenchymal properties of anterior – posterior polarity, migratory and invasive behaviors [1]. This transition is essential during embryonic development, organogenesis, and wound repair. However, EMT may also contribute to the tissue changes observed in diseases such as tissue fibrosis, invasive cancer, rheumatoid arthritis and some other diseases [1]–[7].

Many factors have subsequently been demonstrated to participate in the EMT like behavior since the first inducer, hepatocyte growth factor, was identified in 1985 [8]. These include growth factors and their corresponding cell surface receptors [e.g. transforming growth factor-β (TGF-β), epidermal growth factor (EGF), fibroblast growth factor (FGF)]. Several transcription factors (Snail, ZEB, Twist), and signaling molecules (Wnt, Notch, NF-κB) also contribute to this process [9]–[12]. There has been extensive research detailing of the molecular processes and compositional changes associated with EMT as these could be of value in monitoring in vivo its progression or providing a new approach to regulating these transitions. The loss of E-cadherin expression is a critical and fundamental event in EMT, and many inducers of this process act directly or indirectly by repressing E-cadherin expression [6], [11], [13]–[19]. Increased expression of vimentin and alpha–smooth muscle actin is also associated with EMT in specific cell context [20]–[22]. Although repression of E-cadherin expression in EMT accounts for the loss of intercellular adhesion and polarity, it is still unclear how the cells acquire the capacity of migration [23]–[28].

We recently identified podocalyxin (PODXL) as a markedly up-regulated protein in TGF-β induced EMT of human A549 cells. PODXL is a type I transmembrane glycoprotein and a member of the CD34 family. Similar to other members of this family it can be extensively O-glycosylated and sialylated. Podocalyxin was originally identified on podocytes in kidney where it is essential for normal renal development [29]. It is also expressed by hematopoietic progenitors, vascular endothelia, and a subset of neurons. Podocalyxin has also been observed in subsets of breast, prostate, liver, pancreatic and kidney cancer as well as leukemia [30], [31]. Elevated expression of podocalyxin in these cancers is often associated with aggressive invasion and poor prognosis. Podocalyxin has a number of interaction partners including Na+/H+ exchanger regulatory factor (NHERF), the actin binding protein ezrin, the adhesion molecule L-selectin, and cortactin[20], [32], [33].

Podocalyxin is involved in the regulation of cell adhesion and cell morphology with often seemingly opposing roles. It has an anti-adhesive function in podocytes while it is a pro-adhesive molecule in lymphocytes enhancing their adhesion to immobilized L-selectin [34]–[37]. The latter properties may contribute to the increased rate of cancer cell migration. It is unclear how podocalyxin mediates these distinct effects in different cellular contexts. One suggestion is that the levels of podocalyxin expression may contribute to these apparently contradictory roles in cell adhesion [31]. Low level podocalyxin could establish apical domains and force integrins to the basal surface of cells, thereby enhancing cell adhesion, while increased podocalyxin could strongly induce microvillus formation, depleting basolateral actin and disrupting integrin mediated adhesion.

The present study was initiated to examine the role of podocalyxin in TGF-β induced EMT. Podocalyxin was found to play several roles in EMT like behavior. Its expression was increased following TGF-β treatment and it was required for migration of the transitioned cells. Podocalyxin was also shown to bind and colocalize with secreted collagen type 1. It appears that podocalyxin may play a role in the control of cell migration by regulating the dynamics of cell protrusion formation and interactions with collagen type 1.

## Methods

### Cells and Culture

The human lung adenocarcinoma cell line A549, human embryonic kidney cell line 293T, and human breast cancer cell line MDA-MB-231 were obtained from the American Type Culture Collection (ATCC) (Manassas, VA) and maintained in DMEM supplemented with 10% fetal bovine serum (FBS) (Invitrogen). For induction of EMT, A549 cells were cultured in 10% FBS for 24 hour and then maintained for 72 hours in serum free medium in the presence of 2 ng/ml of TGF-β (Millipore, Billerica, MA).

### Antibodies and reagents

The following antibodies were purchased from Santa Cruz Biotechnology (Santa Cruz, CA): mouse antibodies to human GAPDH, Vimentin, E-cadherin; rabbit antibody to podocalyxin; goat antibody to chain 2 of collagen type 1. A horse radish peroxidase (HRP) conjugated rabbit anti-mouse immunoglobulin was obtained from Sigma-Aldrich (St. Louis, MO). Oregon green 488 labeled phalloidin, cy3 labeled donkey anti-rabbit immunoglobulin, and Oregon green 488 conjugated donkey anti-goat IgG were obtained from Jackson ImmunoResearch (West Grove, PA). A mouse anti-human serum albumen IgG was an in house produced reagent.

### Western Blot

Total cell protein extracts were prepared by lysing cells in buffer containing 100 mM Tris (pH 6.8), 2% SDS, 50 mM DTT and 20% glycerol. The samples were boiled, sonicated and protein contents were estimated using the Pierce bicinchoninic acid assay (Pierce Biotechnology, Rockford, IL). Proteins were separated by electrophoresis on Novex® 4–12% Tris-Glycine gradient Gels (Invitrogen) and transferred to nitrocellulose membranes using a semi-dry transmembrane device (Bio-Rad, Missisauga, ON). The membranes were probed overnight with primary antibody (1 µg/ml) in PBS followed by incubation with the appropriate HRP conjugated secondary antibody as indicated. Amersham ECL Western Blotting reagent was used to develop the blots. The blot intensity was measured by Quantity One software (Bio-Rad).

### Immunoprecipitaton

A549 cells were cultured in four 10 cm dishes for 48 hours to 80% confluence. The media was removed and the dishes were washed with PBS. A total of 250 µl lysis buffer containing 2% Triton X-100 in 50 mM Tris (pH 7.4) 150 mM NaCl, was added to each dish on ice. The cells were collected by scraping and transferred to microfuge tubes. After incubation on ice for 30 minutes, the samples were briefly sonicated and centrifuged at 10,000 rpm for 10 min to remove insoluble debris. The supernatant was collected and aliquots were incubated for 2 hours at 4°C with protein-G Dynal beads pre-coated with 5 µg of monoclonal antibody to either podocalyxin or human serum albumin (HSA). The beads were washed 4 times with lysis buffer and the immunoprecipitates were digested on the beads with trypsin (5 µg/ml) in 100 mM NH_4_HCO_3_. The resulting peptides were analysed by mass spectrometry.

### shRNAmir interference

Three lentiviral clones containing shRNA-mir against podocalyxin (catalog number: RHS3979-98487906, RHS3979-98487914, and RHS3979-98487921) were purchased from Open Biosystems. Lentiviral particles were packaged with vector psPAX2 and vector pMD2.G according to the supplier's instruction (Open Biosystems, Huntsville, AL). A control virus lacking a shRNA insert was used in parallel experiments as control. Virus containing supernatants were transferred to A549 cells at 60% confluence and incubated for 6 hours. Fresh media was added to the cultures and the cells were incubated for 24 hours after which puromycin (5 ng/ml) was added to the cultures to select transduced cells. Puromycin resistant cells were examined for podocalyxin expression. The cells tranduced with shRNA-mir clone RHS3979-98487914 or RHS3979-98487921 produced markedly lower podocalyxin by western blot examination and appeared in obviously changed morphology. A mixed population of puromycin resistant cells from clone RHS3979-98487921 were maintained in media containing puromycin and used in all the experiments. A rescue test was not performed for further demonstration of the specificity.

### Cell migration assay

Transwells (8 µm pore size, 6.5 mm, Corning) were coated with gelatin (0.1%) overnight at 4°C. Cells (2×10^5^/well) in media containing 0.5% serum were added into the inserts and placed in wells containing the same medium plus TGF-β (5 ng/ml) as chemotactic factor. The cells were cultured for 2 to 4 hours and then fixed with either 3.8% paraformaldehyde for immunofluorecence or 30% methanol plus 0.5% crystal violet for quantitation of cell number. The cells remaining on the upper surface of the filter were removed with a cotton swab and the numbers of migrating cells in three high power fields of the lower surfaces of the membranes were counted. A minimum of three wells were counted per experiment.

### Wound healing assay

Confluent cultures of control and silenced A549 cells were maintained in the presence of TGF-β for 48 hours. The monolayers were then scratched with a 200 µl pipette tip and washed to remove the detached cells. The wounded areas were then imaged after incubation for an additional 16 hours in media without TGF-β.

### Immunofluorescence

Cells on chamber slides or transwell filters were fixed with 3.8% paraformaldehyde for 30 min and permeablized with 0.2% triton X-100 for 1 min. The cells were then treated sequentially with the indicated primary antibodies in PBS followed by 3 washes in PBS and subsequent incubation with appropriate fluorescent secondary antibody conjugates. Each of the incubations with antibody was for 30 minutes. Images were collected on a Zeiss Axio Observer microscope with Axio Vision software.

## Results

### TGF-β induced EMT and podocalyxin expression

**Figure 1:**
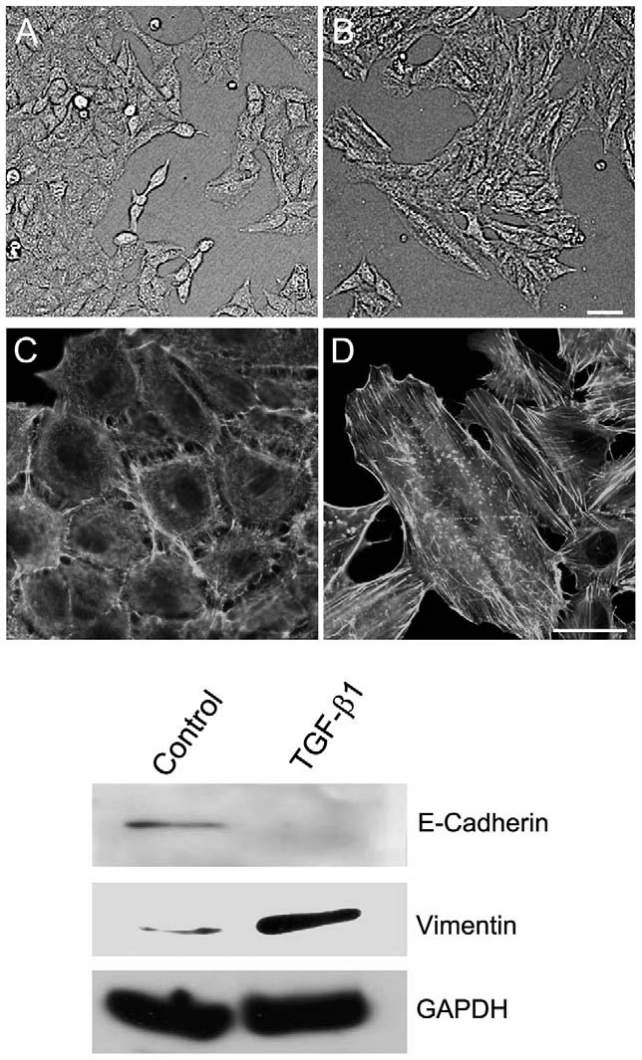
TGF-β induced EMT. A549 cells were cultured in medium without (A, C) or with TGF-β (2 ng/ml) (B, D) for 3 days. Cellular morphology (A, B) was photographed by phase contrast microscope. Actin cytoskeleton (C, D) was visualized by Oregon green 488 conjugated phalloidin staining and photographed by fluorescence microscope. Scale bar = 50 µm. The effects of TGF-β on expressions of EMT markers, E-cadherin and vimentin, were examined by western blot. GAPDH from the same loading was used as control.

Several features of TGF-β treated cells were examined to confirm that EMT like properties were induced under our culture conditions. The A549 cells underwent morphological and compositional changes consistent with EMT following treatment with TGF-β [23], [38]. These included the loss of apical polarity with the acquisition of a more fibroblast like spindle shape and cytoskeletal remodeling with the appearance of actin stress fibers (Fig. 1). There was also a loss of expression of the epithelial marker, E-cadherin, and a concomitant increase of the mesenchymal marker, vimentin.

**Figure 2:**
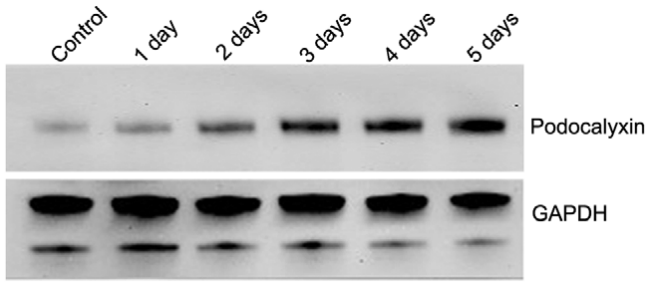
Induction of podocalyxin by TGF-β. A549 cells were incubated with TGF-β (2 ng/ml) for the indicated times and the cells were collected for western blot. The blots were established by mouse anti-podocalyxin and HRP-conjugated rabbit anti-mouse secondary antibody. GAPDH was also monitored on the same filter as control.

In preliminary proteomic studies we observed that PODXL levels increased in A549 cells following TGF-β treatment. The kinetics of these changes was examined by western blot analysis of PODXL levels. There was a modest increase in PODXL following 24 hours treatment. These reached plateau levels following 72 hours treatment and were maintained for at least 120 hours which was the longest time point examined (Fig. 2). The maximal PODXL expression levels at day 3 were approximately 5 times those of control basal. The changes in PODXL expression appeared to precede the development of fibroblast like morphological features as the former were detectable after 24 h of treatment while the latter were only apparent after at least 48 hours of culture with TGF-β [23], [39].

### Podocalyxin is required for cell migration

**Figure 3:**
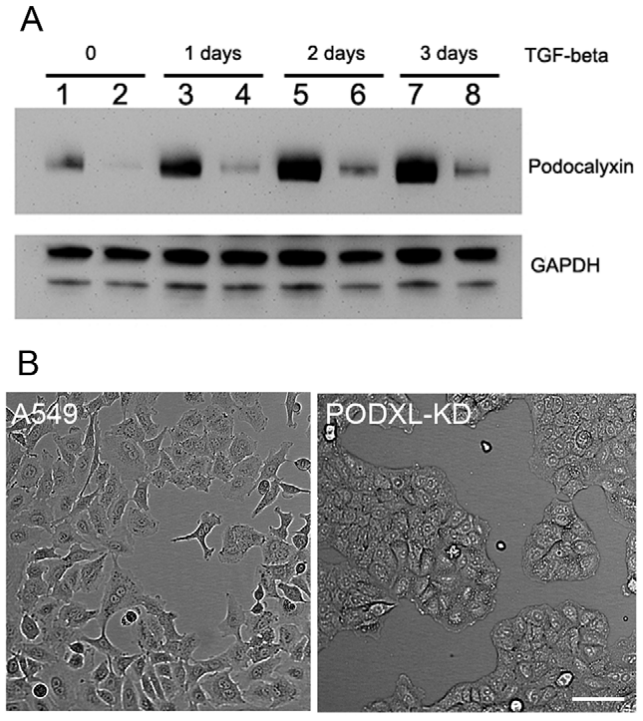
Effects of podocalyxin silence on cell morphology and podocalyxin production. **A**): Podocalyxin shRNA transduced cells (PODXL-KD) (lane 2,4,6,8) and control A549 cells (transduced with the same vector without shRNA insert) (lane 1,3,5,7) were cultured in the presence or absence of TGF-β for 1 to 3 days. Podocalyxin levels were examined by western blot. GAPDH were also determined as loading controls. **B**): Cell morphology under phase contrast microscope before and after silencing podocalyxin. Scale bar = 50 µm.

A characteristic feature of EMT is the acquisition of an invasive migratory phenotype. We questioned whether podocalyxin participated in cell migration in this specific situation. To examine this possibility, we established podocalyxin knock down cell lines (PODXL-KD) using lentiviral vector based shRNAmir silencing. Western blot analysis indicated that podocalyxin levels were reduced to ∼15% of that of control basal levels (Fig. 3A). Following treatment with TGF- β podocalyxin production was increased in PODXL-KD cells but remained at less than 20% of the control cells at all time points (Fig. 3A, compare even and odd lanes at each time point). The PODXL-KD cells became more epithelial in morphology with the formation of tight cell clusters and a reduction in the number of cell protrusions (Fig. 3B).

**Figure 4:**
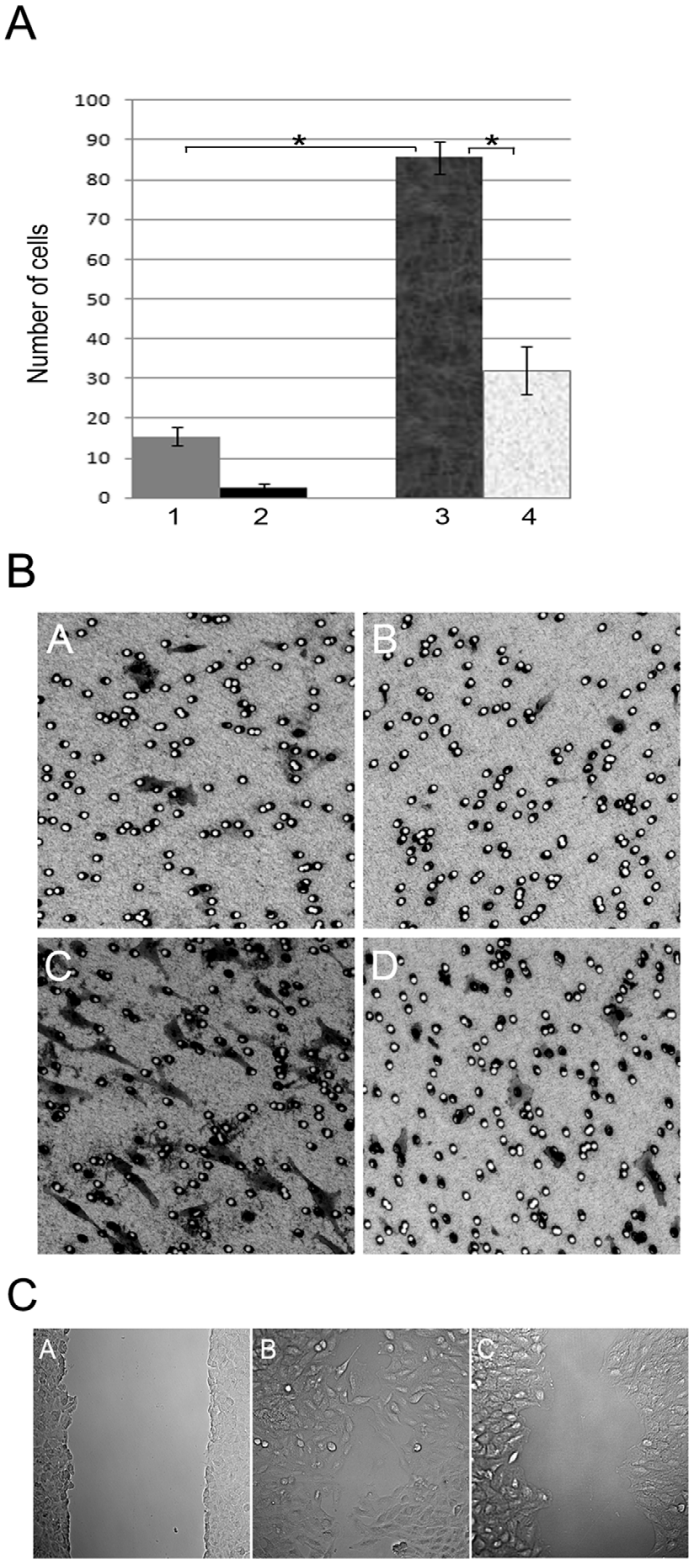
Podocalyxin is involved in TGF-β induced cell migration. **A)** A549 (column 1, 3) and PODXL-KD (column 2,4) cells were cultured for 3 days in medium containing 0.5% serum without (column A,B) or with (column C,D) TGF-β (2 ng/ml). The migration capacities of these cells in response to TGF-β (5 ng/ml) were measured on gelatin coated transwells. The cells were cultured for 4 hours and fixed with methanol containing 0.5% crystal violet. The numbers of cells per 20× magnified lens field were counted (panel A). Three fields per transwell were counted in triplicate experiments. The mean values with standard deviations are indicated on top of each column with a student T test analysis p<0.01 (*). **B**) The morphology of cells that migrated to the lower surface of transwell filters. Images A–D correspond the cells of column 1–4 in panel A. **C**) Wound recovery assay. A549 and PODXL-KD cells were cultured for 48 hours in the presence of TGF-β (2 ng/ml) and were scratched with pipette tip (A). The cells were washed and cultured in medium without TGF-β for 16 hours. The images of cell recovery were taken under phase contrast microscope. A549 (B) and PODXL-KD (C).

Untreated control A549 cells display a low capacity of chemotaxis in response to TGF-β (panel A of Fig. 4). This response was further reduced in PODXL-KD cells (compare columns 1, 2 of Fig. 4A). Pretreatment of control A549 cells with TGF-β for 72 hours induced a marked increase in their chemotactic responses. Although the migration of PODXL-KD cells were also increased by this treatment, they never exceeded ∼30% of treated control A549 cells (column 3, 4). It was notable that there were obvious differences in the morphologies between transmigrated PODXL-KD and control cells (panel B of Fig. 4). TGF-β treated A549 cells passed through the filter and extended large cell projections on the lower membrane surface (C of panel B). In contrast, TGF-β treated PODXL-KD cells entered the membrane pores but very few penetrated and extended to the lower membrane surface (D of panel B).

The migratory capacities of TGF-β treated A549 and PODXL-KD cells were also measured using an in vitro wound repair assay (panel C of Fig. 4). Many TGF-β treated A549 cells migrated into the wound area within 18 hour of wound recovery. These cells exhibited motile shapes with clearly polarized fronts and extensions into the wound area (B of panel C). The situation in the PODXL-KD cells was quite different (C of panel C). There was little evidence of individual cells entering into the wound space. However, the narrowing of the wound space suggests that the cells would eventually close the wound. This could potentially be caused by cell propagation. Collectively these results suggested that podocalyxin is required for TGF-β induced cell migration.

### Podocalyxin levels influence the expression of molecular markers of EMT

The loss of E-cadherin is typically associated with an increase in vimentin expression during EMT. These events are thought to contribute to acquisition of the cellular phenotypic changes associated with the transition. It was therefore questioned whether podocalyxin played any role in regulating these changes.

**Figure 5:**
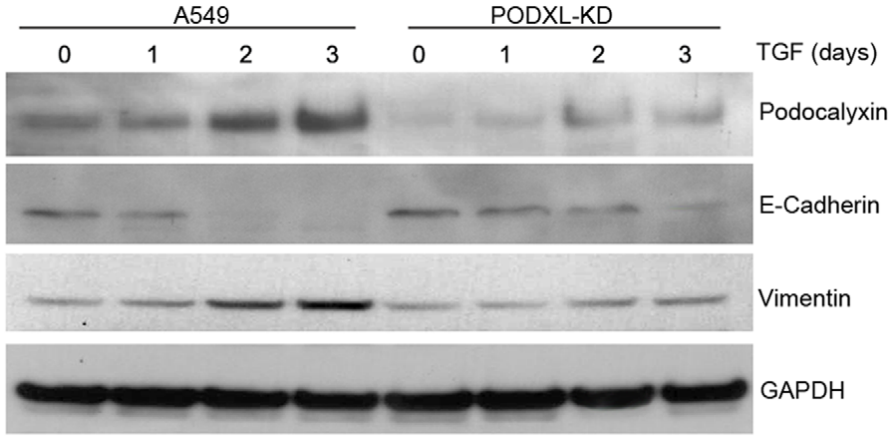
Podocalyxin is required for TGF-β induced EMT marker expressions. A549 and PODXL-KD cells were treated with TGF-β for 1–3 days, and monitored by western blot for their expressions of podocalyxin, E-cadherin and vimentin. TGF-β up-regulated the production of podocalyxin and vimentin in A549 cells, and down-regulated E-cadherin level to zero in a time dependent manner. Silencing of podocalyxin interfered with TGF-β mediated down-regulation of E-cadherin and up-regulation of vimentin.

A549 and PODXL-KD cells were treated with TGF-β for 1 to 3 days and the levels of E-cadherin and vimentin were measured by western blot (Fig. 5). The basal levels of E-cadherin and vimentin were unaffected by podocalyxin silencing despite the clear differences in cellular morphology. Treatment of control cells with TGF-β resulted in the loss of E-cadherin by 48 hours of culture. This was associated with a concomitant increase in vimentin which was most notable by 72 h of culture. However the situation was very different when PODXL-KD cells were treated in a similar fashion. E-cadherin never fully disappeared in PODXL-KD cells following TGF-β treatment. Very modest increases in vimentin were detected from day 2 in PODXL-KD cells. These results suggest that podocalyxin may contribute to the regulation of the molecular changes associated with EMT.

### Podocalyxin affects cell spreading and enriches at the leading edge of migrating cells

**Figure 6:**
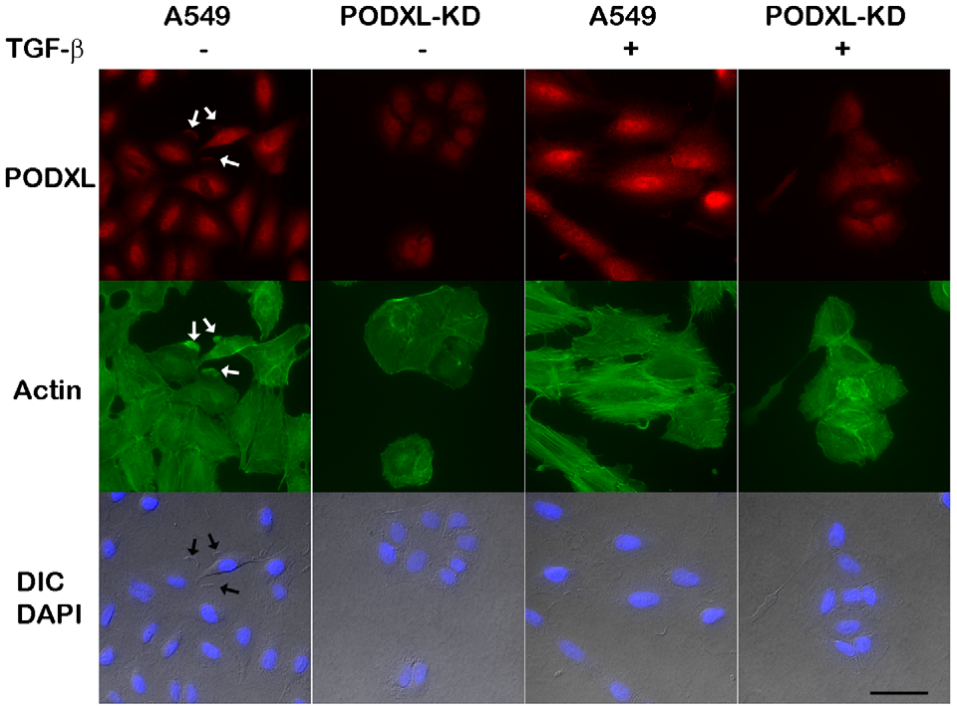
The distribution of podocalyxin. A549 and PODXL-KD were cultured in the presence or absence of TGF-β (2 ng/ml) for 3 days. The cells were fixed with 3.8% paraformaldehyde and permeablized. The expression of podocalyxin (red) was visualized by applying primary rabbit anti-podocalyxin antibody and cy3 conjugated goat anti-rabbit secondary antibody. F-actin was stained with Oregon green conjugated phalloidin. Images of immunofluorescence and differential interference contrast (DIC) were taken for each field and merged. The blue color in the merged pictures indicates nuclei stained by DAPI. Arrows indicated the ruffles of cell protrusions. Scale bar = 50 µm.

As indicated by the results of the previous sections that PODXL plays a role in TGF-β induced EMT and chemotaxis, we examined the expression and distribution of podocalyxin on A549 and PODXL-KD cells in the presence or absence of TGF-β treatment. Podocalyxin appeared in a relatively even and diffuse pattern on un-treated control cells and enriched on ruffle like leading edges of spreading cells (Fig. 6 Arrows in column of A549). Knocking down podocalyxin not only resulted in clear deduction of podocalyxin intensity, but also made the cell smaller and lack protrusions. The cells became aggregated with very few separated single cell in the culture. As E-cadherin was not increased in podocalyxin silenced cells, we deduced that the aggregating pattern was caused by damaged spreading and migrating capacities but not through enhancing cell-cell adhesions. The average number of ruffle with podocalyxin enrichment on A549 cells was 51 based on percentage counting, while only 9 of that were observed on PODXL-KD cells (data not shown). Following TGF-β treatment, the A549 cell changed to a larger spreading shape (2–4 fold in size) with obvious increase in podocalyxin expression and cytoskeleton assembly. The number of ruffle structure markedly decreased in TGF-β treated cells, which could be explained by lacking chemotactic factor gradients, as these cells showed very actively migratory capacities in transwell system. TGF-β slightly enhanced podocalyxin production in PODXL-KD cells and its spreading, but that had never reached the levels of control cells.

**Figure 7:**
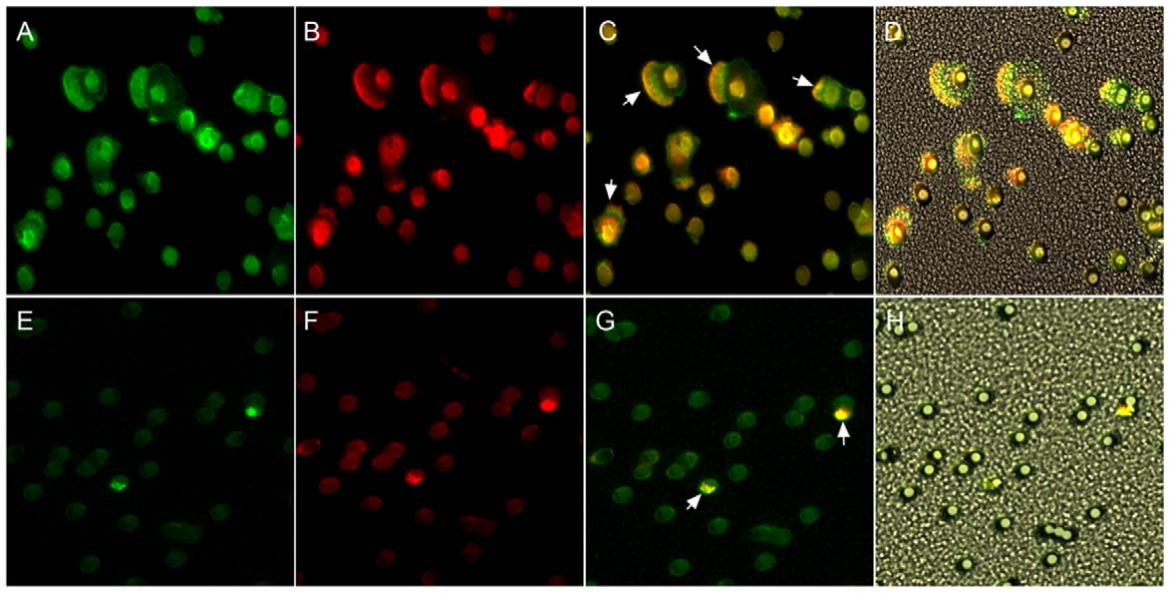
Podocalyxin enriched on the leading edges of migrating cells. A549 (A–D) and PODXL-KD (E–H) cells were pretreated with TGF-β for 3 days and placed in transwell containing TGF-β (5 ng/ml) gradient for two hours. The cells on the filter were fixed with paraformaldehyde (3.8%) and stained for podocalyxin (Cy3 red) and F-actin (Oregon green-488 Phalloidin, green). Both podocalyxin and F-actin enriched on the leading edges of the migrating cells and colocalised each other as showed in merged photo (C). In contrast very few PODXL-KD cells were observed passing through the pores under the same conditions. Bright fields combined images (D and H) were also provided to visualize the locations of pores.

To better determine the distribution of podocalyxin on migrating cells, we treated cells with TGF-β for 72 hours and then induced chemotaxis in transwells against TGF-β. The cells were then fixed and stained for podocalyxin and F actin (Fig. 7). TGF-β induced cells that had passed through the pores of the transwell showed marked podocalyxin enrichment along with F-actin at the leading edges (A-D). In contrast PODOXL-KD cells had very few projections to the lower surface of the transwell (E–H). The few projections observed were stained more intensely for podocalyxin, suggesting that these cells had less inhibition of podocalyxin than the bulk of the non migrating population of cells.

### Podocalyxin associates with collagen type 1

An examination of potential PODXL interacting proteins was initiated as an approach to understanding of the mechanism of podocalyxin actions in regulating cell migration. Anti-podocalyxin derived immunoprecipitates were isolated from A549 cells and analyzed by mass spectrometry. The compositions of these immunoprecipitates were compared with those obtained with a control antibody to human serum albumin (HSA). This provided a basis for identifying proteins that were nonspecifically isolated during the immunoprecipitation process. The selection criteria for candidate proteins were either two peptides matched or log_10_(e) was less than −10, where the log(e) indicates the probability of a random assignment of that confidence level (Table 1). Type I collagen chains and podocalyxin were consistently identified in the anti-podocalyxin immunoprecipitates of all three independent experiments. In contrast other proteins such as keratins, vimentin, plectin and actin were present in both of the podocalyxin and control immunoprecipitates, suggesting that these proteins were not specifically captured during the isolation process. These results suggested that podocalyxin and collagen type 1 are associated with one another in A549 cells.

**Table 1:** Compositional analysis of Podocalyxin containing immunoprecipitates.

			IP of anti-HSA		IP of anti-PODXL	
Accession	Protein ID	MW(kDa)	#pep	log(e)	#pep	log(e)
P63261	Actin	41.8	7	−74.9	7	−68.8
P62807	Histone H2B/a	13.9	3	−21.1	2	−14.2
P04264	Keratin-1	66	12	−119.3	13	−114.8
P35908	Keratin-2	65.4	8	−74.6	6	−50.3
P08729	Keratin-7	51.4	3	−22.4	12	−82
P05787	Keratin-8	53.7	3	−19.5	11	−73.2
P35527	Keratin-9	62	7	−62.7	5	−35.9
P13645	Keratin-10	58.8	7	−65.7	5	−45.8
Q15149	Plectin-1	518.2	9	−67.3	16	−135.2
P08670	Vimentin	53.6	45	−348.1	100	−525.5
Q13148	TDP-43	44.7	3	−33.6		
P62829	RPL-23	14.9			2	−10.5
P02452	COL1A1	138.9			20	−165.9
P08123	COL1A2	129.2			6	−48
O00592	Podocalyxin	58.6			5	−28

# pep: number of peptides matched.

log(e): probability of mismatched.

**Figure 8:**
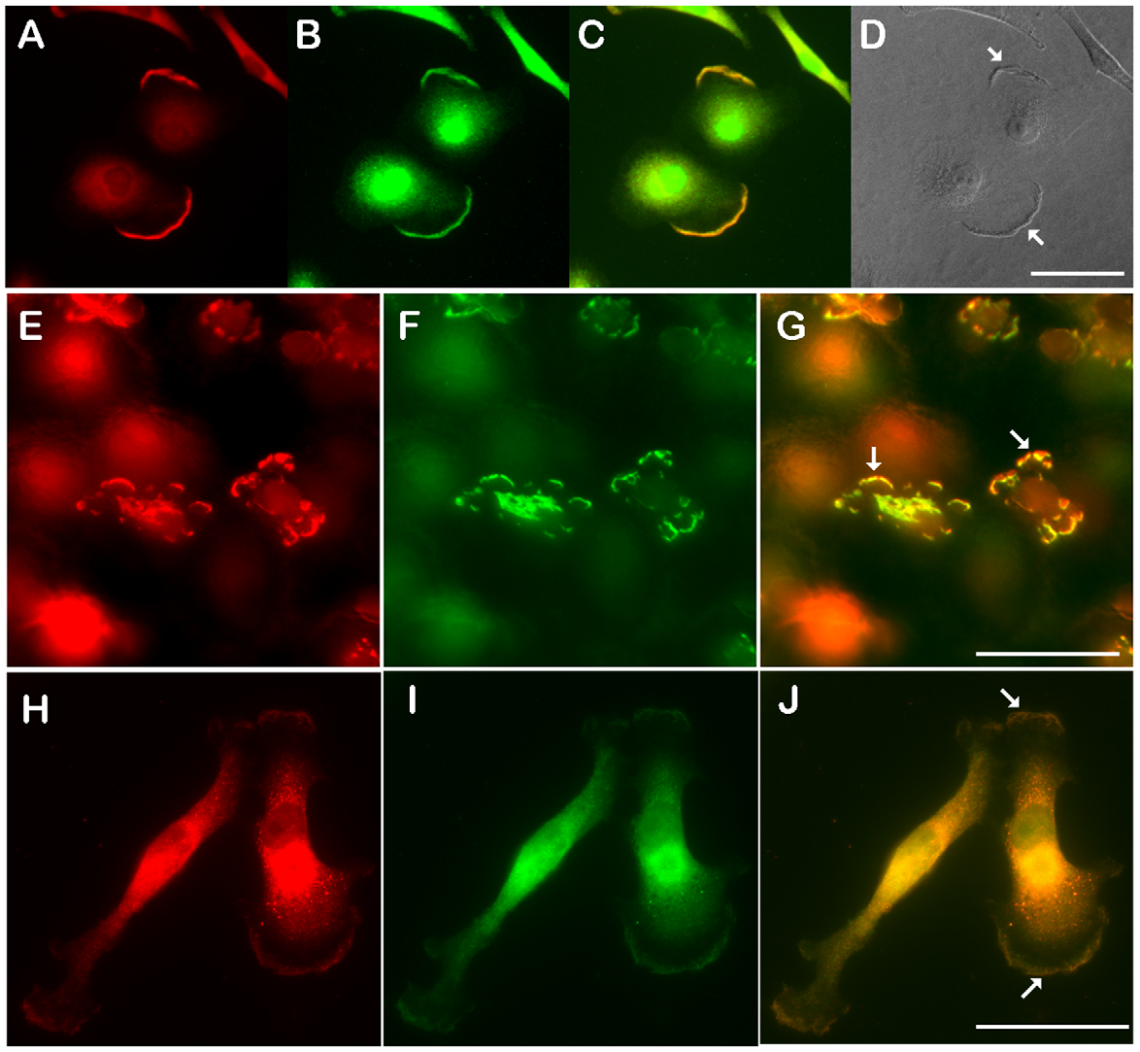
Colocalization of collagen type 1 with podocalyxin. Double immunofluorescence was performed to determine the distribution of podocalyxin (red) and collagen type 1 (green). A549 cells in chamber slide (A–D), A549 cells pretreated with TGF-β for 3 days were transferred into transwells to establishing migration (E–G), and human breast cancer cell MDA-MB-231 in chamber slide (H–J) were fixed and permeablized for following immunofluorescent staining. Rabbit anit-podocalyxin and goat anti-collagen type 1 chain 2 were visualized with Donkey anti-rabbit conjugated with cy3 and Donkey anti-goat conjugated with Oregon green 488. The merged images were also shown respectively (C, G, J). A DIC image (D) was also added to show the ruffle structure. The enrichments and colocalization of both podocalyxin and collagen were indicated by arrows.

Efforts to perform reciprocal immunoprecipitates with three different antibodies to type 1 collagen consistently failed to isolate either collagen or PODXL. Thus we examined the co-distribution of podocalyxin and type 1 collagen alpha 2 chain as an indirect indicator of possible association (Fig. 8). Podocalyxin and collagen type 1 had very similar distribution patterns on A549 cells. There was a marked enrichment and colocalization of collagen and podocalyxin on the edges of cell extending projections. When TGF-β treated cells were exposed to a TGF-β gradient in transwells and then stained for PODXL and collagen, both molecules were seen enriched and co- distributed on the leading edges of projecting cells (Fig. 8).

The distributions of podocalyxin and collagen type 1 in the highly metastatic breast cancer cells MDA-MB-231 were also examined. The majority of these cell population had typical migratory protrusions. In all cases a component of the podocalyxin and collagen were colocalized in the protrusions. These observations suggest that podocalyxin and collagen type 1 interactions may occur during cell migration.

## Discussion

The possible role of EMT like behavior in metastatic cancer has stimulated a number of investigations into the molecular events of this process because information in this area may help to identify new approaches to the treatment [10], [21], [23], [27], [40]. The results of the present study provide several new pieces of information regarding possible podocalyxin function(s) and the process of EMT. These observations are particularly relevant given the observation of podocalyxin on stem cells and cancer cells suggesting potential commonalities in the cellular properties that are involved.

Our results demonstrate that the up regulation of podocalyxin is necessary for epithelial mesenchymal transition. The inhibition of podocalyxin expression prevented the TGF-β induced development of an invasive migratory phenotype and the associated morphological changes. Lowered level of podocalyxin affected TGF-β induced E-cadherin deduction and vimentin increase. This requirement for podocalyxin seemed to be related to responses to TGF-β as the basal levels of vimentin and E-cadherin were comparable in untreated control and PODOXL-KD cells. These coordinated regulations are consistent with a recent report indicating that in pluripotent stem cells podocalyxin interacted with the glucose-3-transporter and the levels of both molecules were coordinately regulated [41]. Collectively these results suggest that podocalyxin is an important component of a regulatory network in TGF-β induced EMT like behavior.

Podoclayxin appears to have an enigmatic role in cell adhesion as it has been described to have both pro and anti adhesive roles depending on cell types and the glycosylation pattern of the molecule [31]. Over expression of podocalyxin in Madin-Darby canine kidney (MDCK) and Chinese hamster ovary (CHO) cell lines have been shown to decrease intercellular adhesion and to reduce the ability of these cells to form appropriate junctional complexes in monolayers [42]. On the other hand, over expression of podocalyxin enhanced cell adhesion to fibronectin and P-selectin [37], [43]. In these cases integrins appeared to be intimately involved in the podocalyxin dependent adhesion. This has led to suggestions that the levels of podocalyxin expression may regulate adhesion by influencing the distribution and activities of other adhesion molecules on the cell surface [30]. Indeed the extracellular domain of podocalyxin was demonstrated to be necessary and sufficient to mediate cell microvillus formation accompanied with actin cytoskeleton recruitment to the polarized membrane region [44]. These observations are consistent with suggestions that podocalyxin may mediate its effects through interactions with other extracellular or membrane proteins. Intracellularly, podocalyxin has been found to form complexes with ezrin and to influence the activities of Rac1, MAPK, and PI3K, increasing the metastatic potential of breast, kidney and prostate cancers [36], [45]. Our results indicate that podocalyxin accumulates at the leading edge of migrating A549 cells in association with arrays of filamentous actin. The silencing of podocalyxin results in a loss of cell migration and a failure of the cells to polarize. These cells are fully adherent to plastic and extracellular matrix proteins suggesting that the loss in migration is not related to an inability to interact with the vessel surface (unpublished data XM, JAW).

The consistent detection of collagen type 1 in immunoprecipitates suggests that these two molecules are associated in some way. It is unclear if this interaction is a direct one or if it is mediated through other elements of a podocalyxin containing complex. As a secretory type protein, collagen is major component of extracellular matrix. We don't know how much collagen was released to extracellular environment during the TGF-β stimulation. To examine the total collagen production, we used three different anti-collagen antibodies to run western blot, but none of them worked.

Integrins are obvious candidates of podocalyxin interacting partners given the effects of antibodies to integrins on podocalyxin dependent adhesion. Preliminary microscopic analysis indicated that α1β1 integrin colocalized with podocalyxin on the leading edge of membrane protrusions (data not shown). However efforts to demonstrate collagen binding integrins such as α1β1 and α2β1 in podocalyxin immunoprecipitates were unsuccessful. While we cannot exclude the possibility that integrins may be associated with collagen-podocalyxin complexes but dissociated during the immunoprecipitation procedure, so we have no data to support such an interaction at this time. Our current data is more consistent with integrin independent interaction of podocalyxin and collagen type 1. However, the interactions between podocalyxin and collagen may determine the distribution of integrins in the processes of cell spreading and migration.

We believe that the demonstration of collagen type 1 on the leading edges and termini of protrusions of migrating cells is a novel observation. These collagen type 1 molecules were directly secreted from cells rather than reorganized from extracellular matrix. The images of migrating cells on transwell suggest that newly synthesized collagen was transported to the cell surface and focused to their protrusions. Collagen type1 is considered to be an important inducer of EMT due to its ability to stimulate E-cadherin adhesion complex disruption by induction of β-catenin phosphorylation [30]. It has also been reported to control cell membrane protrusion formation after remodeling by collagenase-1 on the leading edge of migratory vascular smooth muscle cell [46], [47]. Additionally, soluble collagen type 1 or peptides derived from it have been reported to have chemotactic activities for fibroblasts [48]. It is unclear how the interactions between collagen and podocalyxin would contribute to cell migration.

In summary we have demonstrated that podocalyxin is up regulated during TGF-β induced epithelial mesenchymal transition of A549 cells. Podocalyxin is required for cell migration as it localizes to the leading edges of migrating cells and lowed expression markedly impacts cell movement. Podocalyxin also appears to be intimately associated with the regulation of E-cadherin and vimentin that are involved in effective mesenchymal transition. Another EMT feature, cytoskeleton assembly, was also dependent on the production of podocalyxin. The interactions of collagen and podocalyxin may be important in the development of the invasive phenotype of cells following EMT.
